# Aging exacerbates oxidative stress and liver fibrosis in an animal model of Down Syndrome

**DOI:** 10.18632/aging.205970

**Published:** 2024-06-26

**Authors:** Sebastiano Giallongo, Jessica Ferrigno, Rosario Caltabiano, Giuseppe Broggi, Amer M. Alanazi, Alfio Distefano, Emanuela Tropea, Antonella Tramutola, Marzia Perluigi, Giovanni Li Volti, Eugenio Barone, Ignazio Alberto Barbagallo

**Affiliations:** 1 Department of Biomedical and Biotechnological Sciences, University of Catania, Catania 95124, Italy; 2 Department G.F. Ingrassia, Section of Anatomic Pathology, University of Catania, Catania 95124, Italy; 3 Pharmaceutical Biotechnology Laboratory, Department of Pharmaceutical Chemistry, College of Pharmacy, King Saud University, Riyadh 11451, Saudi Arabia; 4 Department of Biochemical Sciences “A. Rossi-Fanelli”, Sapienza University of Rome, Roma, RM 00185, Italy

**Keywords:** Down Syndrome, oxidative stress, liver, aging

## Abstract

Down Syndrome (DS) is a common genetic disorder characterized by an extra copy of chromosome 21, leading to dysregulation of various metabolic pathways. Oxidative stress in DS is associated with neurodevelopmental defects, neuronal dysfunction, and a dementia onset resembling Alzheimer's disease. Additionally, chronic oxidative stress contributes to cardiovascular diseases and certain cancers prevalent in DS individuals. This study investigates the impact of ageing on oxidative stress and liver fibrosis using a DS murine model (Ts2Cje mice). Our results show that DS mice show increased liver oxidative stress and impaired antioxidant defenses, as evidenced by reduced glutathione levels and increased lipid peroxidation. Therefore, DS liver exhibits an altered inflammatory response and mitochondrial fitness as we showed by assaying the expression of HMOX1, CLPP, and the heat shock proteins Hsp90 and Hsp60. DS liver also displays dysregulated lipid metabolism, indicated by altered expression of PPARα, PPARγ, FATP5, and CTP2. Consistently, these changes might contribute to non-alcoholic fatty liver disease development, a condition characterized by liver fat accumulation. Consistently, histological analysis of DS liver reveals increased fibrosis and steatosis, as showed by Col1a1 increased expression, indicative of potential progression to liver cirrhosis. Therefore, our findings suggest an increased risk of liver pathologies in DS individuals, particularly when combined with the higher prevalence of obesity and metabolic dysfunctions in DS patients. These results shed a light on the liver's role in DS-associated pathologies and suggest potential therapeutic strategies targeting oxidative stress and lipid metabolism to prevent or mitigate liver-related complications in DS individuals.

## INTRODUCTION

Down Syndrome (DS) represents human most frequent aneuploidy, characterized by an extra complete or segment of chromosome 21 (trisomy 21), eventually triggering the dysregulation of several factors [1]. Besides the well-established neurodevelopmental defects and neuronal dysfunction, it has been recently reported how DS individuals also display a plethora of conditions related to unbalanced reactive oxygen species (ROS) production [2]. Such phenotype is dependent on chromosome 21 triplication, triggering the upregulation of proteins involved in redox homeostasis as superoxide dismutase 1 (SOD1), the transcription factor BTB and CNC homology 1 (BACH1), the Protein C-ets-2 (ETS2), carbonyl reductase (CBR), S100 calcium-binding protein B (S100B) [3]. Furthermore, it has been reported that the major regulator of antioxidant response elements (ARE) nuclear factor (erythroid-derived 2)-like 2 (NRF2) is transcriptionally activated in DS individuals [4]. This effect has been reported to be related to the hyperphosphorylation of mitogen-activated protein kinases (MAPKs) which in turn mediates NRF2 phosphorylation thus, promoting its dissociation from Kelch-like ECH-Associating protein 1 (Keap1) but preventing its nuclear translocation [5]. In addition, the high oxidative environment might be responsible for several of the outcomes characterizing DS, including accelerated aging, eventually triggering several of the mental disorders characterizing DS individuals. To this regard, recent studies performed in Ts2Cje DS mouse model [5–7], showed an increased risk of developing a type of dementia mimicking the clinical and pathological course of Alzheimer’s disease (AD) already at the age of 40s [8]. These results are strictly related to the triplication of genes associated with AD such as amyloid precursor protein (APP), β-secretase 2 (BACE2), and S100 calcium binding protein B (S100B) [9], which along with the oxidative unbalance characterizing DS, promote the accumulation of amyloid beta-peptide (Aβ) [3]. Furthermore, the chronic condition of oxidative stress accounts for several comorbidities, including cardiovascular diseases, and certain types of cancer characterizing DS individuals [10, 11]. In this context, liver may also represent one of the organs involved in the complex metabolic impairment of DS, even though liver function in DS patients is poorly described, and the molecular basis involved in the pathophysiological processes remain elusive. To this regard, a vascular portohepatic anomaly in DS individuals has been characterized to impair the direct communication between the right portal vein and the inferior vena cava [12], potentially linking congenital vascular malformation and hepatic vascular shunt [13]. Furthermore, DS individuals show a significant enhanced production of hepatitis B antigen which might be responsible for the occurrence of the autoimmune hepatitis, a chronic and progressive inflammation of the liver from an unknown cause [14]. In addition to this, the alternated levels of several amino acids, along with several metabolites involved in the methylation cycle have been detected in blood samples derived from DS individuals, pointing out metabolic dysfunctions as one of the drivers of DS liver pathologies [15]. Consistently, DS individuals, since childhood, are typically obese and also presenting dyslipidemia and hyperinsulinemia [16]. Previous studies showed that DS patients exhibit a marked trend in developing overweight and obesity, mostly as a result of a lower resting metabolic rate, a higher consumption of energy-rich foods, and a poor physical activity. As a result, these subjects display a higher risk in developing type 2 diabetes, dyslipidemia, hyperinsulinemia, hypertension, and cardiovascular diseases [16]. However, the recent improvement of surgical and early therapeutic intervention in DS morbidities, increased patients’ life expectancy. On the other hand, there exists an enhanced risk of chronic noncommunicable disease as non-alcoholic fatty liver disease (NAFLD), a pathology characterized by the accumulation of fat in the liver, a condition strictly associated to the enhanced insulin resistance reported in DS individuals [15–17]. Furthermore, DS liver is characterized by significative changes in liver morphology showing sinusoidal dilatation, central-vein sclerosis, and portal fibrosis [18]. Finally, other key factors to take into due account are several single nucleotide polymorphisms (SNPs), such as patatin like phospholipase domain containing 3 (PNPLA3), Transmembrane 6 superfamily member 2 (TM6SF2), and Klotho eventually promoting hepatic fat accumulation [19]. Given the correlation standing between DS and NAFLD occurrence, further research is needed to fully understand the pathophysiological mechanisms underlying such association.

## MATERIALS AND METHODS

### Mouse colony

Ts2Cje (Rb(12.Ts171665Dn)2Cje) mice are an established DS murine model displaying a triple copy of a Robertsonian fusion chromosome where the distal end of Chr16 and Chr12 are located. Parental generations were purchased from Jackson Laboratories (Bar Harbour, ME, USA). The mouse colony was raised by a crossbreed of Ts2Cje trisomic females with euploid (B6EiC3SnF1/J) F1 hybrid males (Eu). The parental generations were purchased from Jackson Laboratories (Bar Harbour, ME, USA). These breeding pairs produce litters containing both trisomic (Ts2Cje) and euploid (Eu) offspring. Pups were genotyped to determine trisomy by standard PCR, using Reinoldth’s method [20, 21]. Mice were housed in clear Plexiglas cages (20 × 22 × 20 cm) under standard laboratory conditions with a temperature of 22 ± 2°C and 70% humidity, a 12-h light/dark cycle, and free access to food and water, as previously described [21]. All the experiments were performed in strict compliance with the Italian National Laws (DL 116/92), and the European Communities Council Directives (86/609/EEC). The experimental protocol was approved by the Italian Ministry of Health (#1183/2016-PR). All efforts were made to minimize the number of animals used in the study and their suffering. For this reason, our cohort was composed by 49 mice subdivided as following: 3 months (6 Eu; 6 TS2Cje); 6 months (5 Eu; 6 TS2Cje); 9 months (6 Eu; 6 TS2Cje) and 12 months (6 Eu; 6 TS2Cje). Immediately after isolation, samples were put into liquid nitrogen and then stored at −80°C until utilization.

### GSH evaluation

GSH levels were assayed on tissues homogenized performing a spectrophotometric assay based on the reaction of thiol groups with 2,2-dithio-bis-nitrobenzoic acid at a wavelength of 412 nm (εM = 13,600 M^−1^ cm^−1^, where εM is a wavelength-dependent molar absorptivity coefficient) [22]. Measurements were performed quantified using Synergy H1 (Biotek, Milan, Italy) in quadruplicate per each sample.

### LOOH measurement

Lipid peroxide assay was performed as already reported [22]. Briefly, the reaction is based on the oxidation of Fe^2+^ to Fe^3+^ in the presence of xylenol orange at 560 nm. Measurements were performed quantified using Synergy H1 (Biotek, Milan, Italy) in quadruplicate per each sample.

### RNA extraction and cDNA preparation

Tissue sections were resuspended in 500 mL of PRImeZOL Reagent (#AN1100, Canvax Biotech, Andalusia, Spain). RNA extraction was then performed as previously described [23] and resuspended in RNase-free water. RNA was quantified using Synergy H1 (Biotek, Milan, Italy). 2 μg of RNA from each sample were retro-transcribed using High-Capacity cDNA Reverse Transcription Kit (#4368814; Applied Biosystems; Waltham, MA, USA) according to manufacturer instructions.

### Real time PCR

Gene expression analysis was performed as previously described [24]. As probe, PowerUP SYBR Green Master Mix (#A25742; Applied Biosystem, Waltham, MA, USA) was used. Primers are listed in Table 1.

**Table 1:** Real time PCR primers’ list.

**Gene**	**Forward 5′ --> 3′**	**Reverse 5′ --> 3′**	**Accession number**
*Hmox1*	TGACACCTGAGGTCAAGCAC	CAGCTCCTCAAACAGCTCAATG	NM_010442.2
*Il1β*	TGCCACCTTTTGACAGTGATG	CGTCACACACCAGCAGGTTA	NM_008361.4
*Il10*	GTAGAAGTGATGCCCCAGGC	GACACCTTGGTCTTGGAGCTTATT	NM_010548.2
*Ppar**α***	TGCCTTCCCTGTGAACTGAC	CACAGAGCGCTAAGCTGTGA	NM_001113418.1
*Ppar**γ***	GGTCAGTCATGGAACAGCCA	TTCTGGGAGAGGTCTGCAC	NM_001411509.1
*Fatp5*	TGTAACGTCCCTGAGCAACC	TAAGCCCACATTGCCCTCTG	NM_009512.2
*Cpt2*	GAATGACAGCCAGTTCAGGAAG	GCATGCAGCTCCTTCCCAAT	NM_009949.2
*Col1a1*	CCCTGGTCCCTCTGGAAATG	GGACCTTTGCCCCCTTCTTT	NM_007742.4
*Gapdh*	AACCCTTAAGAGGGATGCTGC	TCTACGGGACGAGGAAACAC	NM_001289726.2

### Western blot analysis

Tissue sections were homogenized for protein extraction as already described [25]. Briefly, a small section of liver was resuspended in 1 mL of phosphate buffer solution (PBS) and then mechanically homogenized by Dounce homogenizer. The suspension was centrifuged 15 minutes at 13000 Rpm and supernatant was collected for further analysis. Therefore, we performed western blot analysis as described in [26]. Rabbit anti heat shock protein 90 (HSP90) (ab59459), heat shock protein 60 (HSP60) (ab46798), and glyceraldehyde-3-phosphate dehydrogenase (GAPDH) (ab8245) were purchased from Abcam (Cambridge, UK). Rabbit anti CLPP (PA5-52722) was purchased from Thermo Fisher Scientific (Waltham, MA, USA). Secondary antibody anti Rabbit-HRP (ab6721) was purchased from Abcam (Cambridge, UK).

### Histopathological analysis

Liver sections were formalin-fixed, paraffin-embedded and treated for histological examination using a standard method [27]. Two pathologists (R.C. and G.B.) separately evaluated all histological slides, blinded to sample identity. The following histological features were assessed on 5 micron-thick sections stained with hematoxylin and eosin and Masson’s trichrome, as previously described [28]: fibrosis, inflammation, steatosis and hepatocellular ballooning. Fibrosis was graded on a 0–3 scale: 0, absence of fibrosis; 1, portal spaces expanded by fibrosis with or without fibrous septa; 2, portal areas expanded by fibrosis with formation of fibrous bridges; 3, numerous fibrous bridges with formation of nodules. The following score was used to grade inflammation: 0, lack of inflammation; 1, periportal inflammation; 2, mild/moderate portal inflammation; 3, marked portal inflammation. Hepatocellular ballooning was scored on a 0–2 scale: 0, absent; 1, mild; 2, moderate/severe. The following 0–3 score was used to quantify steatosis: 0, absent; 1, mild; 2, moderate; 3, diffuse. For each case, the final histological score was performed by summing the scores of fibrosis, inflammation, steatosis, and ballooning.

### Statistics

Data are shown as mean ± standard deviation (SD). Statistical analysis was performed by using Prism 8.0.2. software (GraphPad Software, San Diego, CA, USA). Significant differences were assessed using a one-way ANOVA or the student test, when needed. A value of *p* < 0.05 was considered statistically significant and symbols used to indicate statistical differences are described in figure legends.

## RESULTS

### DS liver displays an enhanced exposure to oxidative stress

**Figure 1:**
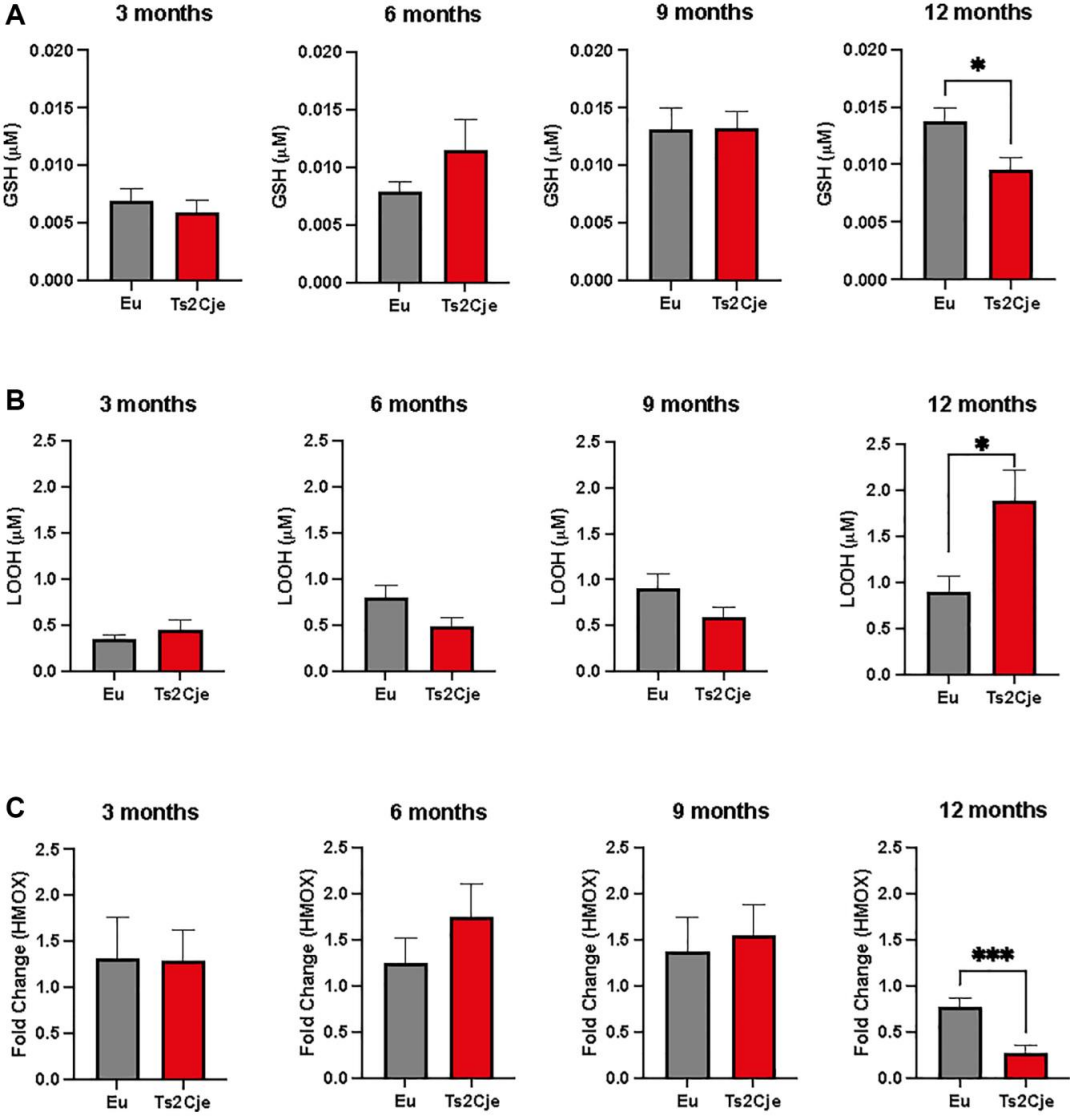
**DS mice are characterized by an increased oxidative stress.** (**A**) GSH levels are decreased in Ts2Cje 12-months old mice liver. Representative scheme for GSH quantitation in Eu and Ts2Cje livers obtained from 3-, 6-, 9-, and 12-months old mice. (**B**) LOOH levels are increased in Ts2Cje 12-months old mice liver. Histograms representative of LOOH spectrophotometric evaluation on Eu and TS2Cje livers obtained from 3-, 6-, 9-, and 12-months old mice. (**C**) HMOX1 expression is downregulated in Ts2Cje 12-months old livers. Representative histogram of the real-time PCR against HMOX1 on the liver extract obtained from 3-, 6-, 9-, and 12-months old Eu and Ts2Cje mice. GAPDH was used as housekeeping gene. Histograms are representative of four different experiments (^*^P ≤ 0.05; ^***^P ≤ 0.001).

DS individuals display an impaired ROS scavenging system, which might be also hampering liver homeostasis [2]. To investigate this outcome also in our mice model, we therefore assayed the accumulation of glutathione (GSH) and lipid peroxide (LOOH) in wild type (Eu) and DS Ts2Cje liver homogenates derived from 3-, 6-, 9-, and 12-months old mice (Figure 1). Our cohort was composed by 49 mice: 3 months (6 Eu; 6 TS2Cje); 6 months (5 Eu; 6 TS2Cje); 9 months (6 Eu; 6 TS2Cje) and 12 months (6 Eu; 6 TS2Cje). Ts2Cje, but not Eu, were characterized by a deficit of GSH at 12-months. Any significant change was evident at any stage before the 12-months (Figure 1A). LOOH levels, on the other hand, were increased in 12-months old Ts2Cje liver homogenates compared to the Eu counterpart (Figure 1B). Similarly to GSH, also LOOH levels were not significatively changed at any of the time points assayed before (Figure 1B). Given this evidence, we next sought to investigate heme oxygenase 1 (HMOX1) expression in 3-, 6-, 9-, and 12-months old mice models by qPCR (Figure 1C). Our results show that there is a trend in increasing HMOX1 expression at 3-, 6-, and 9-months, but it turns to be significatively downregulated at 12-months in Ts2Cje livers compared to Eu counterpart (Figure 1C), overall highlighting an important increase in oxidative stress in DS mice model liver, and an impairment of the antioxidant defenses.

### DS mice livers show an increase of inflammatory markers

**Figure 2:**
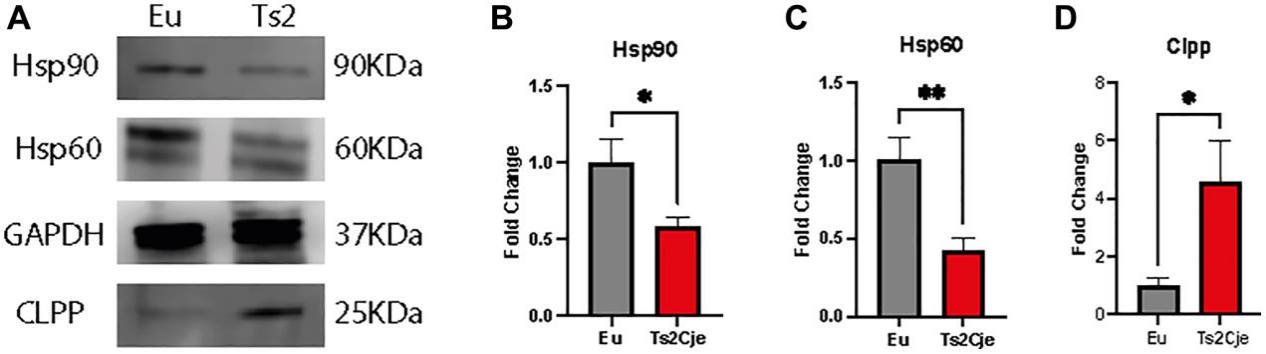
**DS mice show a marked increase in liver inflammation.** Western blot analysis on 12-months old Eu and Ts2Cje liver extracts against Hsp90, Hsp60, and Clpp (**A**). Ts2Cje mice showed a decreased Hsp90 (**B**), Hsp60 (**C**) levels, while Clpp accumulation is increased (**D**). GAPDH was used as housekeeping protein. Histograms are representative of four different experiments (^*^P ≤ 0.05; ^**^P ≤ 0.01).

An increase in oxidative stress as the one we reported in Ts2Cje livers, might be correlated with an increase in the overall inflammatory status [29]. The latter is strictly linked to the accumulation of heat shock proteins (Hsps) [30]. For this reason, we sought to investigate the accumulation of Hsp90 and Hsp60 in our model. Western blot analysis unveiled a marked decrease of both Hsp90 and Hsp60 in Ts2Cje liver protein extract compared to Eu counterpart (Figure 2A–2C; Supplementary Figure 1).

The increased inflammatory status has been reported to also affect the mitochondrial dynamics [31]. In this context, the Caseinolytic Mitochondrial Matrix Peptidase Proteolytic Subunit (Clpp) has been described as one of the major regulators of mitochondrial quality control system [32]. Interestingly, western blot analysis highlighted a marked increase in Clpp protein level in Ts2Cje liver homogenates compared to Eu counterpart (Figure 2A, 2D; Supplementary Figure 1). Overall, these data describe a scenario in which the increase in oxidative stress triggers an inflammatory status activating Hsp machinery and possibly impairing mitochondrial quality system.

### DS liver increases lipid metabolism

**Figure 3:**
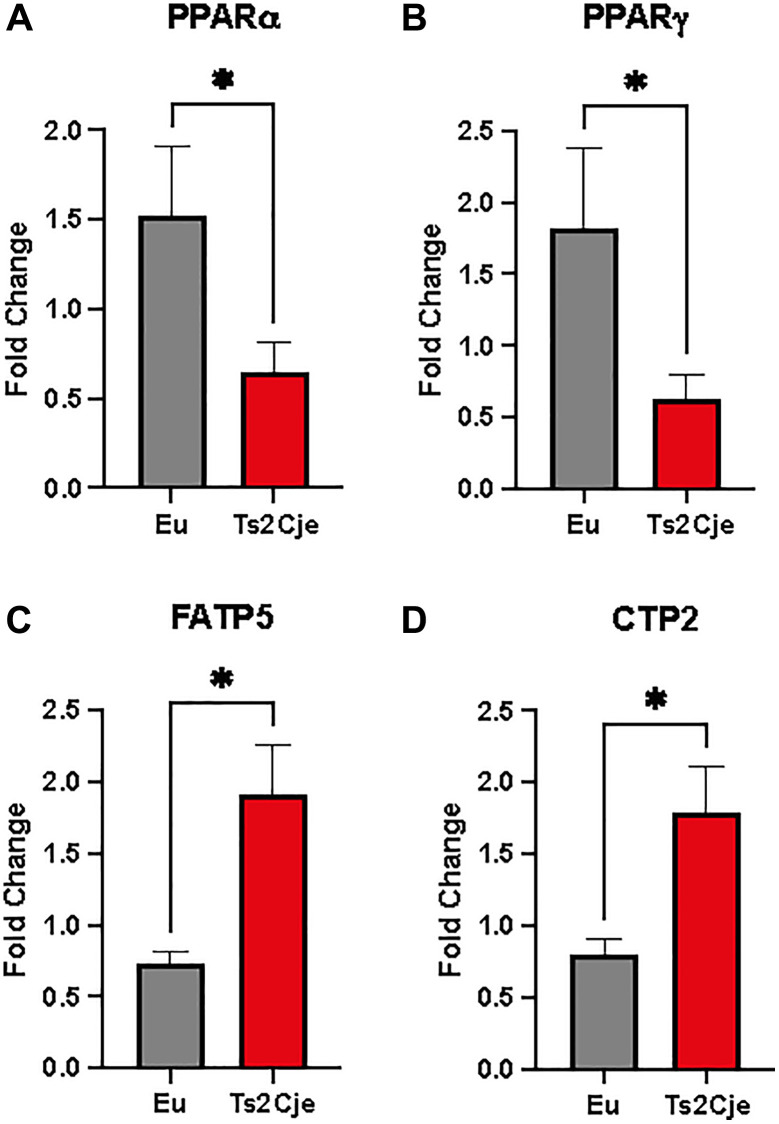
**DS mice have a marked increase in lipid metabolism.** PPARα, PPARγ, FATP5, and CTP2 are differently regulated in Ts2Cje livers. Representative histogram of the real-time PCR against PPARa (**A**), PPARg (**B**), FATP5 (**C**), and CTP2 (**D**) on the liver extract obtained from 12-months old Eu and Ts2Cje mice. GAPDH was used as housekeeping gene. Histograms are representative of four different experiments (^*^P ≤ 0.05).

The decreased level of Clpp has been described to be one of the NASH hallmarks, a pathology strictly associated to liver lipid accumulation [32]. To assay lipid accumulation in our model we assayed the expression of two members of peroxisome proliferator-activated receptors (PPARs) family. Interestingly, the expression of both PPARα and PPARγ in Ts2Cje liver were markedly decreased compared to the Eu counterpart (Figure 3A, 3B). Furthermore, qPCR analysis also described a marked increase in fatty acid transport protein-5 (FATP5) and Carnitine palmitoyl transferase 2 (CTP2) in Ts2Cje, but not in Eu livers (Figure 3C, 3D). Taken together these data we unveil an increase in fatty acids metabolism in DS mice livers.

### DS livers display an increased fibrosis

**Figure 4:**
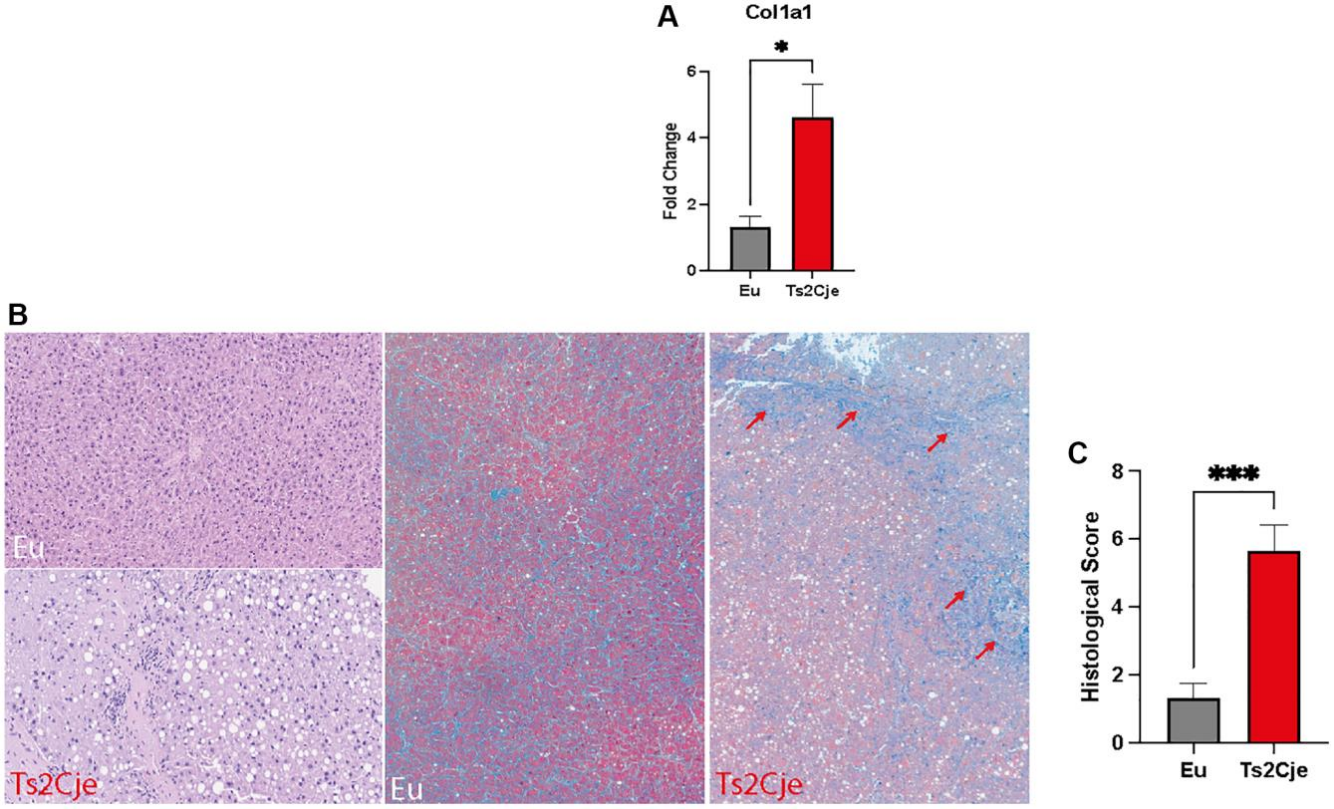
**DS mice show a marked increase in fibrotic markers.** (**A**) Col1a1 expression level is increased in Ts2Cje livers. Representative histogram of the real-time PCR against Col1a1 on the liver extract obtained from 12-months old Eu and Ts2Cje mice. GAPDH was used as housekeeping gene. (**B**) Ts2Cje liver section shows an increased fibrotic level. Representative histological images of the rat livers from control and Ts2Cje group. (Top left panel) Mouse liver from control group showing absence of steatosis (score 0) and mild periportal inflammation (score 1) (hematoxylin and eosin; original magnification 100x). (Bottom left panel) Mouse liver from Ts2Cje group exhibiting moderate steatosis (score 2) and moderate portal inflammation (score 2) (hematoxylin and eosin; original magnification 150x). (Mid panel) Mice liver from control group showing absence of fibrosis (score 0) (Masson’s trichrome; original magnification 50x). (Right panel) Mice liver from Ts2Cje group exhibiting diffuse fibrosis (score 3) with fibrous bridging (arrows) (Masson’s trichrome; original magnification 50x). (**C**) Histological score quantified as in B. Histograms are representative of four different experiments (^*^P ≤ 0.05; ^***^P ≤ 0.001).

The accumulation of lipids within the hepatic tissue might drive the shift from NASH toward cirrhosis [33]. This process is characterized by the accumulation of collagen. To test this outcome, we performed a qPCR testing Collagen Type I Alpha 1 Chain (Col1a1) expression, which was significantly increased in Ts2Cje livers compared to the Eu counterparts (Figure 4A).

Furthermore, we also performed a histological analysis on Ts2Cje and Eu liver sections, evaluating the fibrosis percentage (Figure 4B, 4C). Histologically, Eu group showed lack of fibrosis and hepatocellular ballooning, along with low levels of steatosis and inflammation compared to Ts2Cje counterpart (Figure 4B, 4C). Conversely, the Ts2Cje group fibrosis formation with, at least focal, fibrous septa/bridging and a more widespread inflammation and steatosis than controls (Figure 4B, 4C). Taken together, these data report an increased fibrotic rate in DS liver compared to healthy controls.

## DISCUSSION

Trisomy of chromosome 21 is the genetic signature of DS, impairing the cognitive and intellectual abilities of individuals with this condition. Previous reports showed that DS individuals are characterized by changes also on their metabolic profile [15] eventually affecting the homeostasis of several organs, including liver. The aim of the present study was to study the liver of a DS mouse model, which was previously generated and characterized by Barone and Perlugi’s lab [6, 7, 21, 34]. Interestingly, we found that 12-months old mice liver were characterized by a decreased GSH concentration, a well-known antioxidant molecule [35]. The decreased level of antioxidant defenses prompted us to investigate the concentration of LOOH in this context. Consistently, we also showed a concomitant increased oxidative environment in DS 12-months old mice liver as the result of the possible dysregulation of different factors occurring in redox homeostasis, as recently reviewed by our group [3]. Interestingly, we also reported a significant decrease in HMOX1 expression in 12-months old livers which is consistent with the increased expression of transcription factor Bach1, a master transcription repressor having HMOX1 as one of the main targets [36, 37], reported in the brain of DS subject, and further demonstrated in Ts2Cje mice model by Perluigi’s et al. [34, 36]. In particular, HMOX1 protein plays a role in the complex pathophysiological cascade involved in insulin resistance mechanisms, oxidative stress, metabolic syndrome and cardiovascular diseases [37–39]. The rewire of the oxidative balance in DS livers might also have a crucial role in rewiring the inflammatory response of this organ. Interestingly, we report here a repression of IL-1β transcription, along with an increase of IL-10 expression. As recently reported, an enhancement of IL-10 expression inhibits IL-1β production [40]. This result might be related to the increased susceptibility displayed by DS individuals in the development of several infections, given the lack of an inflammatory response [41]. To further assess the inflammatory response in our model, we also evaluated the protein accumulation of Hsp90, which was decreased in DS-livers. Noteworthy, no significative change in Hsp90 accumulation within DS brains has been reported [42] thus suggesting that such pathway may be specifically involved in the liver of DS. In addition, we also reported a decrease in the protein levels of the mitochondrial shock protein Hsp60. This data is consistent with a previous report describing a deficit in Hsp60 on skin fibroblasts derived from DS individuals [43]. In particular, Hsp60 is the major mitochondrial HSP, in charge for preventing protein aggregation following ROS unbalancing [44]. Therefore, we sought to assess the protein accumulation of one of the major regulators of mitochondrial fitness, Clpp, which we found to be upregulated in our DS model. Interestingly, it has been reported that under high fat diet, Clpp downregulation correlates with an increased protection against obesity and hepatic steatosis, also preventing insulin resistance [45, 46]. Furthermore, Clpp is also part of the machinery preventing hepatocytes senescence [45]. Therefore, we here hypothesize that the upregulation of this protein might be linked to the increased insulin resistance extensively described in DS individuals [15]. Furthermore, it might also be part of a compensatory effect preventing cellular senescence and liver cirrhosis, typical of DS individuals. To further investigate such possibility, we also assessed the expression level of PPARα and PPARγ, two ligand-activated transcription factors part of the nuclear hormone receptor superfamily, in turn in charge for regulating adipogenesis and insulin resistance [47]. Interestingly, the downregulation of PPARα and PPARγ, such as the one we highlighted in our model, could be related to the accumulation of lipid droplets within hepatocytes, also contributing to the insulin resistance reported in DS individuals [48, 49]. The accumulation of lipid droplets in DS liver is further supported by our data showing an increased expression of FATP5 and CTP2 in 12-months old liver compared to the wild-type counterpart. The former is responsible for the uptake of long-chain fatty acids [50]. Consistently, its expression has been reported to be inversely correlated to NAFLD progression [51]. The increase in fatty acids uptake might also be correlated to increased CTP2 expression, in turn in charge for initiating fatty acids oxidation eventually promoting their clearance [52]. In this context, fatty acids accumulation might work as a driver for the onset of liver fibrosis. Consistently, we reported an enhanced expression of Col1a1 in 12-month-old DS mice liver. Since an increased Col1a1 is usually associated with an enhanced fibrotic rate in hepatocellular carcinoma [53], we also decided to assess fibrosis of wild-type and DS livers by histopathology. Interestingly, our results corroborated a scenario in which DS livers display higher fibrosis and hepatocellular ballooning, together with increased steatosis and inflammation. Overall, these data unveil a significant increased risk for DS individuals to develop liver pathologies. Interestingly, liver failure has been reported to DS newborns showing transient abnormal myelopoiesis, a pathology characterized by transient appearance of blast cells and eventually also affecting liver homeostasis [54]. Furthermore, a diffuse lobular fibrosis around proliferating ductular elements and residual hepatocytes, as the one characterizing our DS models, was reported in DS newborns individuals presenting a severe liver disease [55]. These effects are further enhanced by the higher obesity rate reported in DS individuals, in turn promoting NAFLD onset by accumulation of hepatic fatty acids [56].

Overall, our work unveils a scenario in which DS liver is characterized by an impaired ROS scavenging system resulting in a significant impairment of redox homeostasis and leading to an impaired response to inflammatory stimuli. Finally, such results correlated with the increased fibrosis of DS animals, along with the accumulation of fatty lipids. In conclusion, our results put the basis for the use of antioxidants supplementation in DS patients to prevent liver-associated pathologies.

## Supplementary Materials

Supplementary Figure 1
