# Supplementary material for: Aging exacerbates oxidative stress and liver fibrosis in an animal model of Down Syndrome
Source: Aging (Albany NY). 2024 Jun 26;16(12):10203–15. doi: 10.18632/aging.205970 (PMC11236314; doi:10.18632/aging.205970)
Supplement: Supplementary Figure 1 [file aging-16-205970-s001.pdf]

SUPPLEMENTARY FIGURE

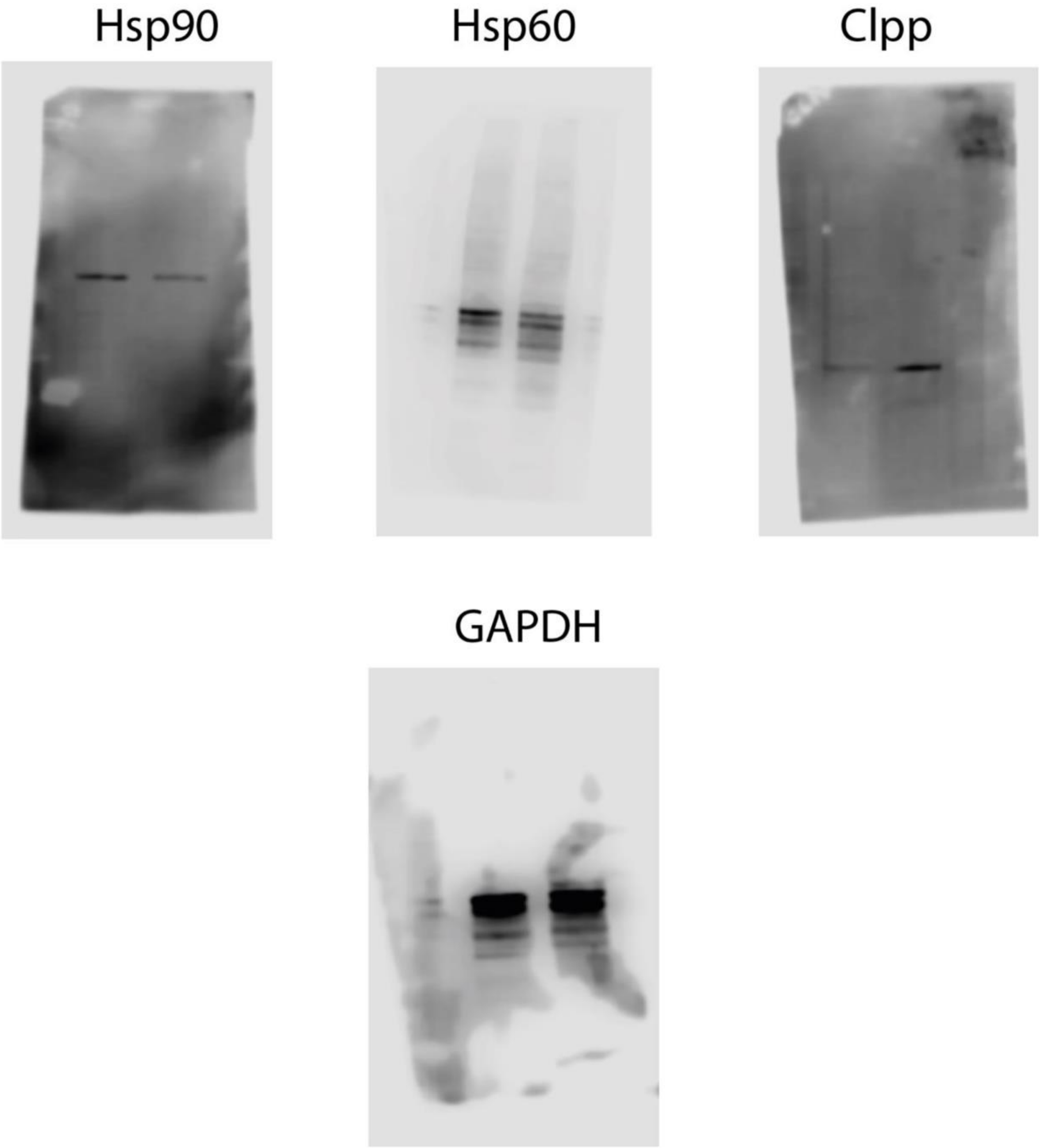

Supplementary Figure 1. Western blot analysis on Hsp90, Hsp60, GAPDH and Clpp. Full membranes derived by the western blot analysis on Hsp90, Hsp60, GAPDH and Clpp.
